# Clinical outcomes of artificial meniscus scaffolds for partial meniscus injury: a systematic review and meta-analysis

**DOI:** 10.1186/s43019-025-00293-2

**Published:** 2025-09-30

**Authors:** Afsaneh Jahani, Mohammad Hossein Ebrahimzadeh, Mohsen Dehghani, Maedeh Sharafoddin, Ali Moradi, Fateme Nikbakht, Nafiseh Jirofti

**Affiliations:** 1 Orthopedics Research Center, Department of Orthopedic Surgery, Mashhad University of Medical Sciences, P. O. Box 91388-13944, Mashhad, Iran; 2 Bone and Joint Research Laboratory, Ghaem Hospital, Mashhad University of Medical Sciences, Mashhad, Iran; 3 Faculty of New Sciences and Technologies, Department of Biomedical Engineering, Semnan University, Semnan, Iran; 4 Department of Regenerative Medicine and Cell Therapy, Emam Reza Hospital, Mashhad University of Medical Sciences, Mashhad, Iran; 5 Clinical Development Research Unit, Ghaem Hospital, Faculty of Medicine, Mashhad University of Medical Sciences, Mashhad, Iran; 6 Department of Epidemiology, School of Health, Mashhad University of Medical Sciences, Mashhad, Iran

**Keywords:** Partial meniscus injury, Collagen meniscus implant, Poly urethane based scaffolds, Meta-analysis, Systematic review

## Abstract

**Background:**

Meniscal injuries, involving damage to the critical fibrocartilaginous structure of the knee joint, often necessitate surgical intervention, including meniscal allograft transplantation or the use of commercial implants. Despite advances in implant based therapies, there is no consensus regarding the comparative efficacy of collagen meniscus implants (CMI) versus polyurethane-based (PU-based) scaffolds. This review aimed to systematically evaluate and compare the clinical outcomes associated with these two implant types for partial meniscal repair.

**Methods:**

A comprehensive systematic review was conducted to evaluate the clinical outcomes of meniscal implants by searching multiple databases including Medline/PubMed, Web of Science, Embase, Scopus, and Cochrane in the temporal range of 1999–2024. The review focused on pre–post studies and assessed various patient-reported outcome measures, including the visual analog scale (VAS), international Knee Documentation Committee (IKDC), Lysholm, knee injury and osteoarthritis outcome score (KOOS), as well as the Tegner activity score. These outcomes were evaluated across different follow-up periods [short-term (6 month to 2.5 years), mid-term (2.5–5 years) and long-term (10 years)] following meniscal implant implantation. A random-effects meta-analysis model was used to address heterogeneity, along with a sensitivity analysis to evaluate the robustness of pooled estimates. The National Institutes of Health (NIH) quality assessment tool was utilized to assess the methodological quality in the studies.

**Results:**

The meta-analysis identified 26 studies that met the inclusion criteria, and the overall quality of the included studies was mostly fair to good. The analysis showed that both CMI and PU-based scaffolds improved clinical outcomes in patients with partial meniscus injuries, with the implants evaluated across short-term, mid-term, and long-term follow-up periods. Specifically, the analysis found: the VAS scores significantly improved during the short-term follow-up by an average of −1.86 points for CMI and −1.98 points for PU-based scaffolds. Lysholm scores significantly improved at short-term follow-up, increasing by an average of 29.26 points for CMI and 24.98 points for PU-based scaffolds. For the Tegner score, CMI implants showed an average increase of 2.02 points in the short-term, while PU-based implants exhibited a negligible change of −0.05 points.

**Conclusions:**

Both CMI and PU-based scaffolds demonstrated improved clinical outcomes, but showed some differences in effectiveness over follow-up periods. PU-based scaffolds offer faster integration and short-term effectiveness, while CMI promotes gradual tissue regeneration and long-term stability. Although these differing characteristics support personalized meniscal repair strategies, the lack of comparative studies limits definitive clinical guidance.

Level of evidence: Level III, IV.

**Supplementary Information:**

The online version contains supplementary material available at 10.1186/s43019-025-00293-2.

## Background

Meniscus, as a semilunar pad of cartilage, plays a vital role in optimizing force transmission and providing stability, load distribution, and shock absorption in knee and joint protectors [1]. Meniscus damages are multifactorial causes which happen owing to several mechanisms that depends on the amount tearing of the cartilage tissue [2–4]. In recent years, a range of treatments has been employed to address minor meniscus injuries, which are categorized into three primary approaches: non-surgical treatments, meniscectomy, and meniscus repair. Evidence indicates that nonsurgical treatment is particularly effective in the short term and can also be successful in cases involving arthritis. Partial meniscectomy may be beneficial for tears located in the avascular white-white zone. Consequently, meniscus repair techniques and tissue engineering using artificial implants have gained attention as viable treatment options. The use of artificial implants for partial meniscus injuries is a relatively new approach aimed at regenerating or replacing damaged tissue [5]. Currently, commercially available implants include the Collagen meniscus implant (CMI), and polyurethane-based (PU-based) scaffolds such as NUsurface, and Actifit [6]. CMI is a natural polymer-based implant derived Achilles tendon collagen type I, enriched with glycosaminoglycans (GAGs) such as chondroitin sulfate and hyaluronic acid [7, 8]. In contrast, NUsurface and Actifit are synthetic polymer-based implants. Actifit consist of an 80% polycaprolactone (PCL), 20% polyurethane and polycarbonate-urethane polyester composite, and includes ultrahigh molecular weight polyethylene (UHMWPE) reinforcement fibers [9, 10]. Actifit also incorporates ultrahigh molecular weight polyethylene (UHMWPE) reinforcement fibers. NUsurface is made of medical-grade polycarbonate urethane (PCU) [11]. The clinical use of implants has yielded variable outcomes, complicating the establishment of definitive conclusions regarding their efficacy and optimal selection. Nevertheless, PU-based biodegradable implants have demonstrated promising tissue ingrowth and favorable histological results [12]. Several studies report significant clinical improvements over a mid-term follow-up, with implant durability estimated at around 87.6% [13]. CMI similarly shows safety and satisfactory clinical outcomes with low failure rates. Both PU and CMI implants generally yield good clinical results, including significant symptom relief and functional improvement, alongside relatively low failure rates, supporting their use in appropriate clinical trials [14]. However, neither implant type has shown superiority over meniscectomy in terms of cartilage protection. The current evidence is limited by a lack of high-quality, long-term comparative studies. Reported failure rates vary, with PU-based scaffolds demonstrating failure rates between approximately 7% and 18%, and CMI between about 6.5% and 7%, depending on follow-up duration [15]. Imaging studies of PU-based scaffolds have documented cases of partial resorption and implant shrinkage, indicating challenges in maintaining implant integrity over time. Consequently, the absence of robust randomized controlled trials (RCT) with extended follow-up and rigorous methodology restricts definitive assessment of meniscal implant effectiveness and durability. Further systematic, comparative research is necessary to identify the most effective implant types and optimize treatment strategies for partial meniscal defects.

### Aim

This study is a comprehensive systematic review and meta-analysis aimed at identifying the most effective commercial implant for partial meniscus injuries on the basis of evaluating its clinical outcomes in both short-term (6 months to 2.5 years), mid-term (2.5–5 years) and long-term (10 years) follow-ups. This review critically appraised the literature and sought to identify potential sources of possible heterogeneity between studies to inform the best treatment options for patients with partial meniscus injuries. Accordingly, we evaluated knee and general scores including visual analog scale (VAS), International Knee Documentation Committee (IKDC), Lysholm, knee injury and osteoarthritis outcome score (KOOS) (subscales: activities of daily living (ADL), symptoms, sports, pain, and quality of life (QOL), as well as the Tegner activity following the CMI and PU-based scaffolds). On the basis of the best knowledge of our authors and the comprehensive primary search, last systematic reviews focused on two implant including CMI and Actifit, without conducting a meta-analysis [16]. Our systematic review and meta-analysis specifically assess commercial scaffolds made from collagen and PU polymers for the treatment of partial meniscal injuries, with the goal of offering valuable insights for clinicians.

## Methods

The review protocol was prospectively registered on the International Prospective Register of Systematic Reviews (PROSPERO) database under the identification number CRD42023459314, and direct link. Accordingly, the systematic review was conducted in adherence to the Preferred Reporting Items for Systematic Reviews and Meta-Analyses (PRISMA) guidelines and checklist.

### Search strategy

This systematic review and meta-analysis evaluated the clinical studies in English which adhered to the PICOD strategy. The population (P) included study arms that evaluated the patients with partial meniscus injuries who received the intervention (I) either a CMI or PU-based (NUsurface & Actifit) meniscal scaffolds. There were no control (C) group assignees as the interventional groups were introduced as study arms. Including articles’ study design (D) was identified as pre–post (before–after) clinical studies. In addition, the excluded data included other arms of the clinical trials that did not assess the preferred interventions.

**Fig. 1:**
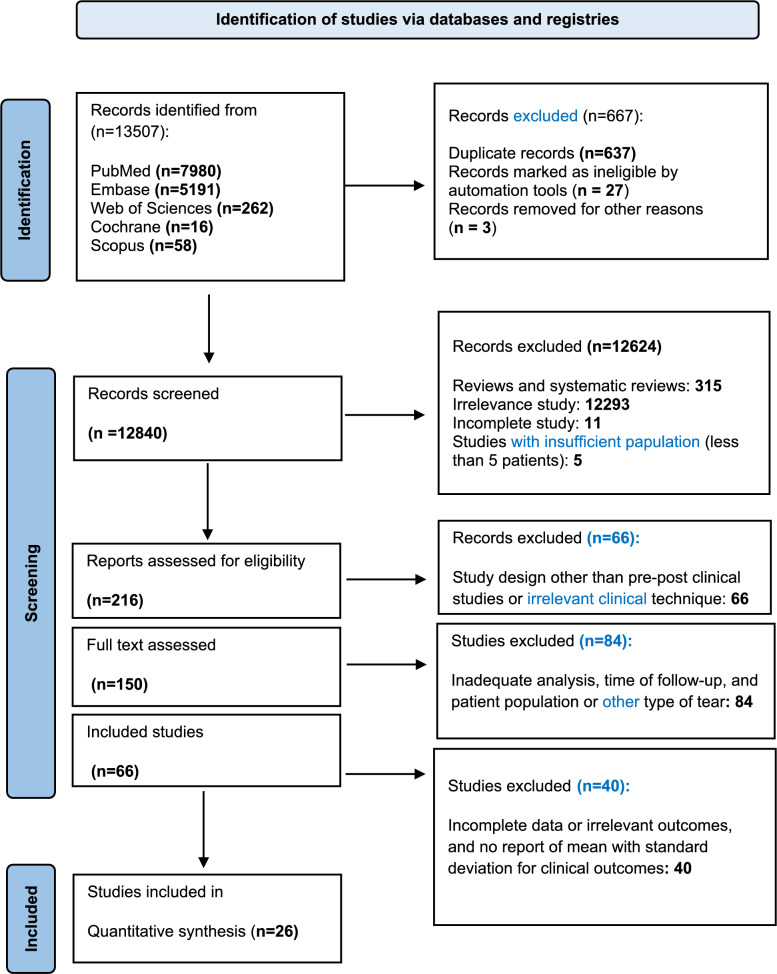
PRISMA flowchart for selection and screening of studies

The search of studies was conducted in May 2024 according to the PRISMA principles and the search period was from 1999 to 2024. Web of Science, PubMed, Embase, Scopus, and Cochrane library databases, as well as grey literature (Google scholar and ProQuest), were searched for studies related to outcomes following implantation with the CMI, NUsurface, and Actifit. The search strategy included terms such as “partial meniscal lesion” and “artificial scaffolds.” All references were searched using the following keywords and MeSH terms in topics including ((“partial meniscus repairing”) OR (“partial meniscectomy”) **AND** (“meniscal scaffold*”) OR (“Biosynthetic scaffold*”) OR (“artificial scaffold*”) OR (“CMI implant”) OR (“Actifit implant”) OR (NUsurface). Figure 1 illustrates a flowchart depicting the study selection process for qualitative and quantitative data synthesis.

### Eligibility criteria

The present systematic review included pre–post clinical studies, evaluating the treatment of partial meniscus defects using artificial implants. Also, Eligible study designs include pre–post clinical studies, as well as registry-based investigations. Studies must provide studies are based on type of implants, follow-up period, defect size (measured in cm^2^), and precise location of the defect within the meniscus in addition to demographic and general data. Evaluation of clinical scores including VAS, IKDC, Lysholm, KOOS with its subscales for ADL, symptoms, sports, pain, and QOL, as well as the Tegner activity following the CMI and PU-based scaffolds will be interest as critical/important clinical outcomes. Studies with incomplete data, irrelevant outcomes, reviews, letters, editorials, technical notes, case reports, case series with fewer than five patients, observational studies, and biomechanical studies were excluded from the analysis.

### Data extraction and outcome measurement

Two authors (A.J and M.S) conducted the selection of articles and extracted the relevant data independently, and any disagreements were resolved through mutual discussion. Subsequently, title and abstracts were screened based on the inclusion and exclusion criteria. Data extraction included demographic information such as the first author, country, year of publication, type of study, sex, mean age, body mass index (BMI), sample size, type of implants used, average defect size, defect location, clinical scores, and follow-up period. Extracted clinical outcomes included VAS, IKDC, Lysholm, KOOS, and Tegner activity scores. A detailed study description is presented in Table 1, 2 (see Supplementary File 1).

We analyzed studies that reported clinical outcomes as means with standard deviations (mean ± SD) and adjusted data from other studies with different reporting indexes to ensure consistency in our analysis. Regarding the adjustment of data, for studies that reported standard errors (SE), confidence intervals (CI), or ranges instead of SD, SDs were calculated using established formulas. These conversions followed the recommendations outlined by the Cochrane Handbook for Systematic Reviews of Interventions [17] and following the methods outlined by Furukawa et al. and Wan et al. [18, 19].

Additionally, to ensure consistency and accuracy, all patients within a group had to complete the minimum follow-up period to be included in the analysis.

## Quality assessment

The National Institutes of Health (NIH) as a quality assessment tool was employed to assess the methodological quality and risk of bias of studies. Using 12 critical question scale of included pre–post clinical studies without a control group, focusing on the treatment of patients using artificial meniscal implants. Each question is answered with either “yes,” “no,” “not applicable (NA),” or “not reported (NR).’ The quality of each study categorizes by using tool based on the score, which can be poor (less than 5), fair (5–8), or good (more than 8). The tool facilitated a comprehensive assessment of various domains, including confounding biases, selection methodologies, and deviations in interventions. Two researchers, A.J and M.S, conducted a dual and independent application of this checklist to evaluate the included studies. In case of any disagreements and conflicts, the opinion of the third researcher (N.J) was regarded as the final decision.

### Statistical analysis

The current meta-analysis aimed to assess the clinical outcomes of patients with partial meniscus injuries which treatment by either CMI or the NUsurface and Actifit device. Regarding the mentioned scores, reported means, SD, and sample sizes from each included study were analyzed. Forest plots were utilized to visualize heterogeneity and to calculate the pooled weighted mean difference (WMD) and standardized mean difference (SMD) along with the corresponding 95% CI. According to the observed clinical and methodological heterogeneity, a random-effects meta-analysis was performed to account for the variability in the study populations [20]. Cochran’s Q statistic was used to assess the statistical significance of heterogeneity. Additionally, the I^2^ statistic was employed to quantitatively assess the degree of heterogeneity among the included studies. To determine the impact of individual studies on heterogeneity and the reliability of the summary results, a sensitivity analysis was performed by systematically excluding each study [21]. Given the significant heterogeneity observed, subgroup analyses were conducted based on implant type, follow-up period, and demographic characteristics. Furthermore, potential publication bias was thoroughly examined through careful visual inspection of funnel plots and statistical tests, including Begg’s and Egger’s tests, to ensure the robustness of the findings. All statistical tests were two-tailed, with a significance level set at less than 0.05 for all analyses except for the heterogeneity test. Stata version 17.0 (Stata Corp., College Station, TX) was utilized for all statistical analyses [22].

## Results

### Studies selection and characteristics

In total, 13,507 studies were identified thorough comprehensive search across all databases. After removing duplicates and incorrect citations, 12,870 records remained and were screened independently by two reviewers (A.J. and N.J.). Following the removal of duplicates and irrelevant references, 12,840 records underwent title and abstract screening, which yielded 216 potentially eligible studies for full-text review. Overall, 66 studies were excluded after screening and found to have inadequate study designs or used irrelevant clinical techniques, which contrasted with our eligibility criteria. Subsequent evaluation of the full texts resulted in the identification of 150 studies that met the predefined inclusion criteria. Studies were excluded at this stage if they reported insufficient patient numbers, lacked follow-up time, inadequate data and analyses, and no report of mean with standard deviation for clinical outcomes. Finally, 26 pre–post clinical studies were included in the present systematic review and meta-analysis. Figure 1 displays a PRISMA flow diagram for selection of studies [23–46]. Main characteristics of the 26 included studies are presented in Table 1 (see Supplementary File 1).

### Quality assessment findings

In total, 26 studies were included for the quality assessment using the NIH Tool. Most studies demonstrated clear research objectives and appropriate statistical analyses, yet common issues such as loss to follow-up and lack of blinding were appointed as limitation parameters. The group level intervention for all the studies were un-applicable. Out of the 26 studies, 19 (68%) met the majority of the criteria and were scored as “good” quality, 2 (7%) studies were scored as “poor” quality due to major limitations, such as insufficient methodological descriptions, inadequate sample sizes, and incomplete reporting of results. The remaining five (19%) studies were scored as “fair” quality, typically lacking in one or two key areas such as participant selection clarity or outcome measure reliability. The detailed results of this appraisal are presented in Table 3 (see Supplementary File 1), providing a comprehensive overview of the strengths and weaknesses identified across the included studies.

### Visual analog scale (VAS)

**Fig. 2:**
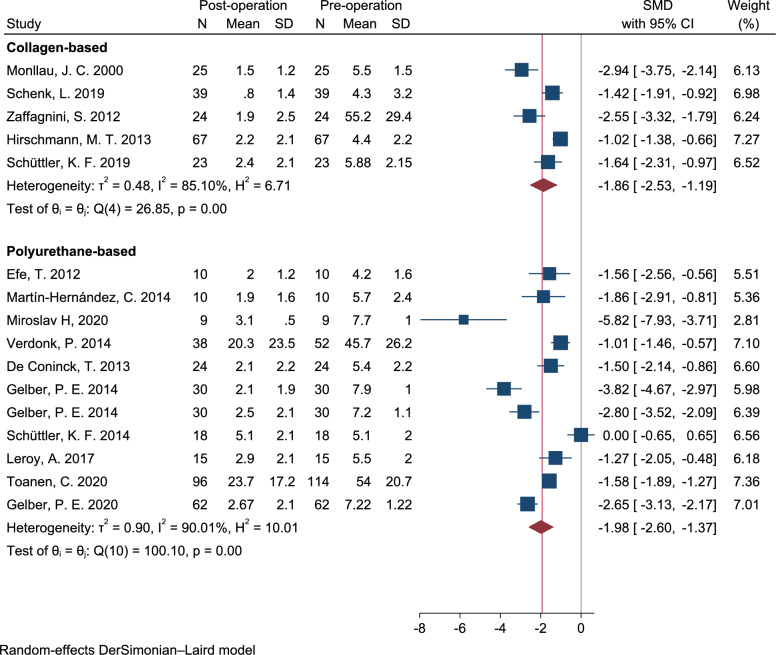
Forest plot of the standardized mean difference (SMD) on postoperative Visual Analog Scale (VAS) in patients undergoing meniscal implant implantations (CMI and PU-based). Diamond represents the summary standardized mean difference (pooled SMD) estimate and its width shows corresponding 95% CI with random effects estimate. I^2^ test and Cochran’s Q statistic were used to assessing the statistical heterogeneity (*P* < 0.10) across studies. Q (df): Cochran’s Q statistic test and degree of freedom (df); H^2^: Ratio of total variance to within-study variance; *I*^2^ (%): Percentage of total variation across studies due to heterogeneity; T^2^ (tau-squared): Between-study variance estimated in a random-effects model; θ (theta): Overall pooled effect size estimation.)

**Fig. 3:**
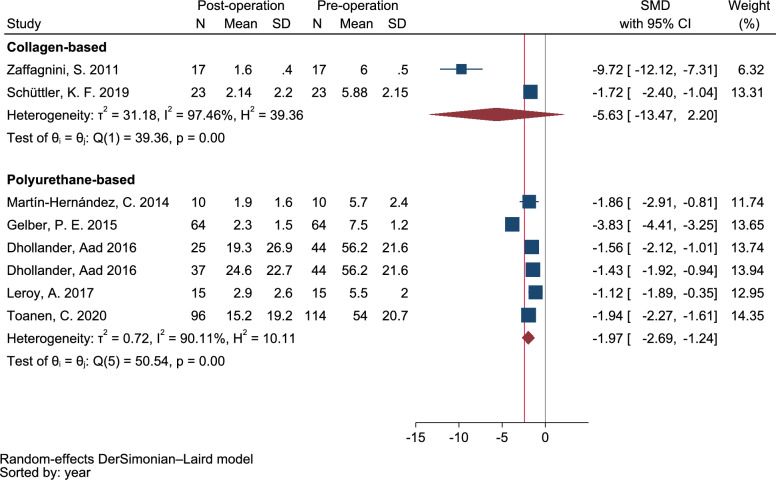
Forest plot of the standardized mean difference; SMD on postoperative visual analog scale (VAS). Diamond represents the summary standardized mean difference (pooled SMD) estimate and its width shows corresponding 95% CI with random effects estimate. I^2^ test and Cochran’s Q statistic were used to assess the statistical heterogeneity (*P* < 0.10) across studies. Q (df): Cochran’s Q statistic test and degree of freedom (df); H^2^: Ratio of total variance to within-study variance; *I*^2^ (%): Percentage of total variation across studies due to heterogeneity; т^2^ (tau-squared): Between-study variance estimated in a random-effects model; θ (theta): Overall pooled effect size estimation.)

**Fig. 4:**
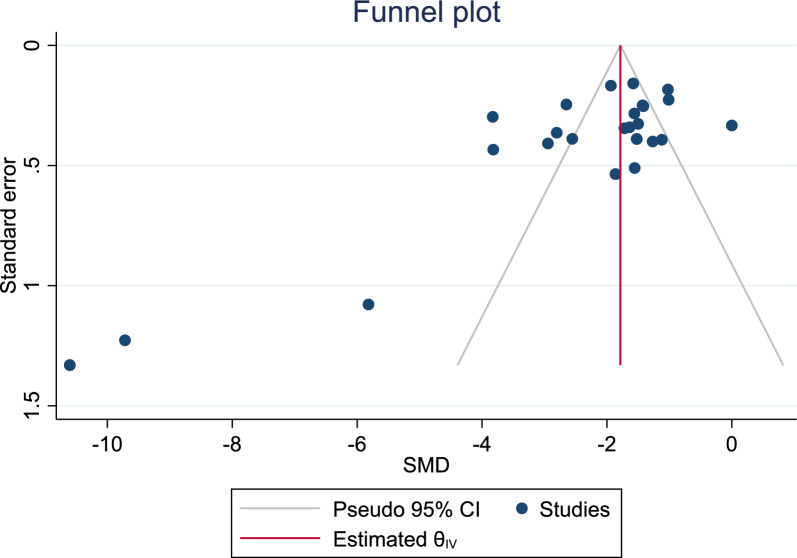
Begg’s funnel plot for assessing the presence of publication bias

A meta-analysis of twenty six clinical trials, including both pre- and post-intervention studies, confirmed a significant improvement in pain scores during the short-term follow-up period by an average of −1.86 points [95% CI −1.19 to −2.53, *I*^2^: 85.2%, Q: 26.8 (*p* < 0.001)] for CMI. The use of PU-based scaffolds for partial meniscus injury treatment resulted in a mean score of −1.98 [95% CI −1.37 to −2.60, *I*^2^: 90.0%, Q: 100.1 (*p* < 0.001)]. Additionally, in the mid-term follow-up period, the average pain scores were −1.97 [95% CI −1.24 to −2.69, *I*^2^: 90.1%, Q: 50.5 (*p* < 0.001)] for PU-based scaffolds and −5.63 [95% CI −13.47 to 2.20, *I*^2^: 97.4%, Q: 39.3 (*p* < 0.001)] for CMI implants. Our obtained results indicated that the meta-analysis model was robust based on the sensitivity analysis of short-term and mid-term follow-up which revealed a consistent mean change in VAS scores ranged of summary standardized mean differences (SMDs) from −2.05 to −1.79 and from −2.63 to −1.93, respectively. The forest plot, shown in Figs. 2, 3 illustrates the pooled SMDs estimate and its corresponding 95% CI with random effects estimate for short and mid-term follow-ups. The high I^2^ value of 85.10% indicated a high degree of heterogeneity among the reported data for the VAS score. Additionally, there was no evidence of publication bias, as shown both visually and through statistical tests. Figure 4 showed the Begg’s funnel plot for assessing the presence of publication bias. SMD of VAS was plotted against the precision of the study.

### International knee documentation committee (IKDC) score

A meta-analysis of pre–post clinical studies evaluating the IKDC score confirmed a significant improvement following treatment with PU-based scaffolds, with a WMD of 31.25 points [95% CI −22.24 to −40.27, *I*^2^: 91%, *Q*: 100.8 (*p* < 0.001)] at short-term follow-up. Similarly, the IKDC score improved by 11.60 points (95% CI 5.48–17.72) in patient treated with CMI during the same period. The robustness and reliability of these findings were confirmed through sensitivity analyses, which demonstrated consistent mean changes in IKDC scores for PU-based scaffolds within a narrow WMD range of 28.51–32.89. At mid-term follow-up, the meta-analysis revealed a continued significant improvement in IKDC scores, with a by 33.00 points (95% CI 26.20–39.81, *I*^2^: 82.4%, *Q*: 34.1 (*p* < 0.001)) for PU-based scaffolds and 14.30 points (95% CI 4.56–24.04) for CMI. Sensitivity analysis confirmed the consistency of these findings, with summary WMDs ranging from 32.02 to 34.98, indicating the robustness meta-analysis model.

### Lysholm score

**Fig. 5:**
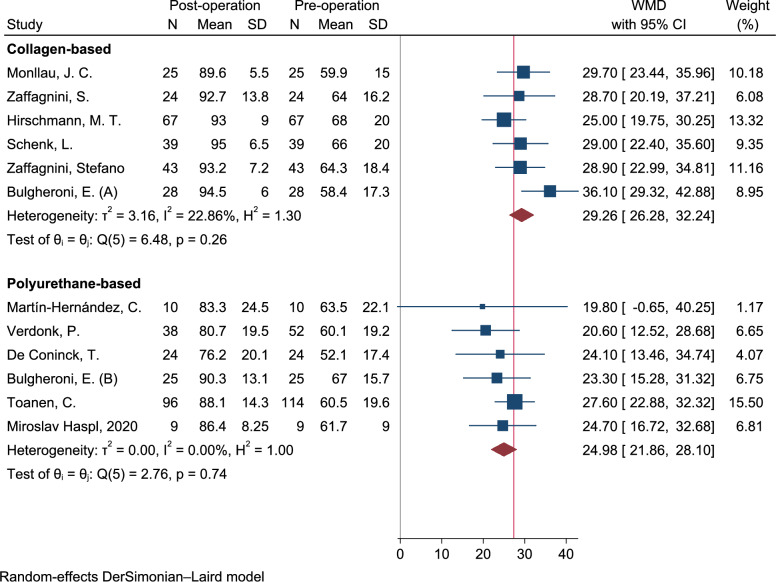
Forest plot of the weighted mean difference; WMD on postoperative Lysholm score. (Q(df): Cochran’s Q statistic test and degree of freedom (df); H^2^: Ratio of total variance to within-study variance; I^2^ (%): Percentage of total variation across studies due to heterogeneity; т^2^ (tau-squared): Between-study variance estimated in a random-effects model; θ (theta): Overall pooled effect size estimation.)

As shown in Fig. 5, the meta-analysis of pre–post clinical studies demonstrated a significant short-term improvement in Lysholm scores following treatment with both CMI and PU-based scaffolds. Specifically, the WMD was 29.26 points [95% CI 26.28–32.24, *I*^2^: 22.8%, *Q*: 6.4 (*p* = 0.26)] for CMI and 24.98 points [95% CI 21.86–28.10, *I*^2^: 0.0%, Q: 2.7 (*p* = 0.74)] for PU-based scaffolds. Sensitivity analysis confirmed the robustness and consistency of these findings, with WMD ranges of 27.94–30.47 for CMI and 22.94 to 25.75 for PU-based scaffolds, indicating a stable and reliable meta-analytic model. For mid-term follow-up, the Lysholm score improved by 46.10 points (95% CI 42.93–49.27) after CMI treatment and by 27.77 points (95% CI 21.48–34.07) following PU-based scaffold implantation. At long-term follow-up, the improvements remained substantial, with a WMD of 33.67 points [95% CI 22.94–44.40, *I*^2^: 91.7, Q: 36.1 (*p* < 0.001)] for CMI and 50.70 points (95% CI 47.71–53.69) for PU-based scaffolds. For mid-term follow-up, the Lysholm score improved by 46.10 points (95% CI 42.93–49.27) after CMI treatment and by 27.77 points [95% CI 21.48–34.07, *I*^2^: 28.9, Q: 4.2 (*p* = 0.24)] following PU-based scaffold implantation. At long-term follow-up, the improvements remained substantial, with a WMD of 33.67 points (95% CI 22.94–44.40) for CMI and 50.70 points (95% CI 47.71–53.69) for PU-based scaffolds.

### Tegner activity score

**Fig. 6:**
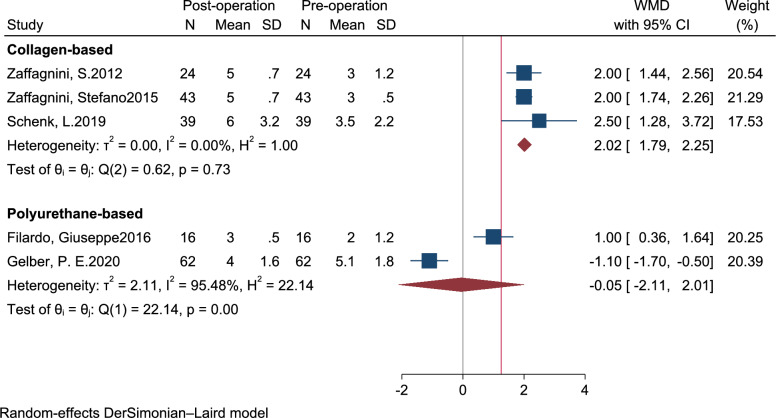
Forest plot of the weighted mean difference; WMD on postoperative Tegner score

Three pre–post clinical studies were included in meta-analysis of Tegner activity scores. As shown in Fig. 6, during short-term follow-up, CMI implants led to a mean score improvement of 2.02 points [95% CI 1.79–2.25, *I*^2^: 0.0%, *Q*: 0.62 (*p* < 0.001)], indicating a significant enhancement in activity levels. In contrast, PU-based scaffolds showed a negligible change, with a mean difference of −0.05 points [95% CI −2.11 to 2.01, *I*^2^: 95.48%, *Q*: 22.14 (*p* < 0.001)], suggesting no statistically significant improvement during the same period. In the mid-term follow-up, the Tegner score increased by 3.00 points (95% CI 2.70–3.30) for CMI implants and by 1.41 points [95% CI 0.05–2.76, *I*^2^: 70.5, *Q*: 3.4 (*p* = 0.07)] for PU-based scaffolds, indicating a moderate recovery of activity levels for both interventions, with a more pronounced effect observed in the CMI group. Long-term outcomes showed improvement with both CMI and PU-based meniscal scaffolds, with greater improvement observed in the CMI group. Specifically, the Tegner score improved by 2.98 points [95% CI 2.71–3.24, *I*^2^: 0.0%, *Q*: 0.62 (*p* = 0.73)] for CMI implants and by 1.50 points (95% CI 0.82–2.18) for PU-based scaffolds.

### Knee injury and osteoarthritis outcome score

#### Activities of daily living

A meta-analysis of nine pre–post clinical studies evaluating CMI use demonstrated a pooled mean improvement in ADL scores of 31.85 points (95% CI 22.10–41.59). For PU-based scaffolds, the mean ADL score was 11.60 points [95% CI: 5.48–17.72, *I*^2^: 79.9%, *Q*: 29.87 (*p* < 0.001)] in the short-term follow-up. Analysis of four pre–post clinical studies on PU-based scaffolds showed a mean ADL score of 21.63 points [95% CI 16.65–26.62, *I*^2^: 11.21, *Q*: 3.38 (*p* = 0.34)] for mid-term follow-up. Sensitivity analysis demonstrated a consistent mean change in ADL scores (range of summary WMDs: 20.31–22.75), suggesting that the meta-analysis model employed was robust.

#### Symptoms

Our meta-analysis revealed the following symptoms scores for short-term follow-up: CMI had a mean symptoms score of 3.40 points (95% CI −2.11 to 2.01), while PU-based scaffolds had mean symptoms score of 19.10 points [95% CI 12.54–25.65, I^2^: 68.8%, Q: 16.03 (*p* = 0.01)]. Sensitivity analysis showed a consistent mean change in symptoms scores range of summary WMDs: (16.59, 21.70), indicating robustness of the meta-analysis model for PU-based scaffolds. For mid-term follow-up, the symptoms score was 24.60 points (95% CI 13.49–35.71) for CMI and 18.35 points [95% CI 11.07–25.63, I^2^: 66.6%, Q: 12.01 (*p* = 0.02)] for PU-based scaffolds. Sensitivity analyses, conducted to assess the robustness of the meta-analytic model, revealed a stable range of WMDs, varying between 20.31 and 22.75. This consistency across different model assumptions supports the reliability of the findings.

#### Sports

The analysis showed a significant improvement in sports scores following the use of PU-based scaffolds, with a pooled mean increase of 32.13 points [95% CI 23.46–40.79, *I*^2^: 71.7, *Q*: 21.2 (*p* < 0.001)] in the short-term follow-up and 28.26 points [95% CI 11.22–45.30, *I*^2^: 90.1%, *Q*: 30.5 (*p* < 0.001)] in the mid-term follow-up. Sensitivity analysis demonstrated a consistent change in Sports scores, with the range of summary WMDs 28.26 to 32.133, indicating the robustness of the meta-analysis model for PU-based scaffolds. Similarly, CMI implants were associated with a significant increase in the sports score, with a mean improvements of 23.50 points (95% CI 18.25–46.75) in the short term and 36.90 points (95% CI 23.53–50.27) in the mid-term follow-up. These findings suggest that both PU-based scaffolds and CMI contribute to improved sports performance post-intervention, with slightly higher mid-term gains observed for CMI.

#### Pain

A meta-analysis of pre–post clinical studies indicated that the mean improvement in pain score was 25.32 points (95% CI 15.75–34.89) following treatment with CMI, and 24.35 points [95% CI 19.83–28.88, *I*^2^: 27%, *Q*: 8.22 (*p* = 0.22)] for PU-based scaffolds. Sensitivity analysis showed a consistent mean change in pain scores (range of summary WMDs: 22.97, 25.40), demonstrating the robustness of the meta-analysis model for PU-based scaffolds. Furthermore, at mid-term follow-up, pain score improved significantly, with a mean increase of 26.80 points (95% CI 14.50–39.10) following CMI implantation, and 26.30 points [95% CI 18.72–33.88, *I*^2^: 56.5%, *Q*: 6.9 (*p* = 0.08)] following treatment with PU-based scaffolds.

#### Quality of life

An analysis of seven pre–post clinical studies revealed that, during short-term follow-up, the mean improvement in QOL scores was 25.92 points [95% CI 20.50–31.34, *I*^2^: 48.5%, *Q*: 11.66 (*p* = 0.07)] following treatment with PU-based scaffolds. Additionally, CMI treatment resulted in a mean QOL scores improvement of 21.10 points (95% CI 9.91–32.29). Sensitivity analysis showed a consistent range of WMDs for PU-based scaffolds (24.51, 27.74), indicating that the meta-analysis model of PU-based scaffolds was robust. Furthermore, at mid-term follow-up, QOL scores continued to improve significantly, with a mean increase of 26.90 points (95% CI: 13.73 to 40.07) for CMI and 31.38 points [95% CI 17.89–44.87, *I*^2^: 89%, *Q*: 27.34 (*p* < 0.001)] for PU-based scaffolds (Table 1).

**Table 1:** Summary of results for the patient reported outcomes

Outcomes		CMI scaffold			PU-based scaffold			Follow-up
		Number of studies	Nonathletes WMD (95% CI)	*I*^2^ (%)	Number of studies	Nonathletes WMD (95% CI)	*I*^2^ (%)	
IKDC		1	11.60 (5.48–17.72)	NA	10	31.25 (22.24–40.27)	91.07	Short-term
		1	14.30 (4.56–24.04)	NA	7	33.00 (26.20–39.81)	82.42	Mid-term
Lysholm		1	46.10 (42.93–49.27)	NA	4	27.77 (21.48–34.07)	28.95	Mid-term
		4	33.67 (22.94–44.40)	91.70	1	57.70 (47.71–53.69)	NA	Long-term
Tegner		1	3.0 (2.70–3.30)	NA	2	1.41 (0.05–2.76)	70.57	Mid-term
		3	2.98 (2.71–3.24)	0	1	1.50 (0.82–2.18)	NA	Long-term
KOOS	ADL	1	11.60 (5.48–17.72)		9	31.85 (22.10–41.59)	91.79	Short-term
		0	–	–	4	21.63 (16.65–26.62)	11.21	Mid-term
	Symptoms	1	3.40 (−4.26–11.06)	NA	6	19.10 (12.54–25.65)	68.82	Short-term
		1	24.60 (13.49–35.71)	NA	5	18.35 (11.07–25.63)	66.69	Mid-term
	Sports	1	32.50 (18.25–46.75)	NA	7	32.13 (23.46–40.79)	71.70	Short-term
		1	36.90 (23.53–50.27)	NA	4	28.26 (11.22–45.30)	90.19	Mid-term
	Pain	1	25.32 (15.75–34.89)	NA	7	24.35 (19.83–28.88)	27.01	Short-term
		1	26.80 (14.50–39.10)	NA	4	26.30 (18.72–33.88)	56.51	Mid-term
	QOL	1	21.10 (9.91–32.29)	NA	7	25.92 (20.50–31.34)	48.55	Short-term
		1	26.90 (13.73–40.07)	NA	4	31.38 (17.89–44.87)	89.03	Mid-term

*CMI* collagen meniscus implant, *PU* polyurethane, *KOOS* knee injury and osteoarthritis outcome score, *IKDC* international knee documentation committee, *ADL* knee injury and osteoarthritis outcome, *QOL* Quality of life; mid-term: 6 months to 2.5 years; long-term: 10 years

## Discussion

The present systematic review and meta-analysis evaluated the use of CMI and PU-based scaffolds for the treatment of partial meniscal injuries. The findings indicate that both types of meniscal implants can significantly improve clinical outcomes during follow-up periods. Meniscus preservation is a crucial aspect of joint surgery, as it aims to delay or prevent the need for total knee replacement [47]. The evolution of meniscal treatment dates back to 1874, when meniscal reinsertion was first described as an alternative to meniscectomy [48]. Meniscectomy, while effective for symptom relief, increases peak contact pressure within the knee and reduces joint lubrication, contributing to progressive cartilage degeneration and the eventual development of osteoarthritis (OA). To address these adverse outcomes, meniscal allograft transplantation was introduced over 30 years ago [49]. However, owing to limitations such as restricted access to skeletal muscle tissue banks and stringent regulations on allograft use in certain countries, the use of artificial implants has been proposed as a novel treatment for partial meniscus injuries [9].

Among these, the CMI, a porous collagen-GAG matrix composed of purified type I collagen isolated from bovine Achilles tendons, has been widely studied. PU-based meniscal scaffolds are synthetic, biodegradable implants with a reported degradation period of 4–6 years and are utilized in the treatment of meniscus injuries [5]. The main finding of previous studies shown that both CMI and PU-based scaffolds significant improvement in clinical outcomes over long-term follow-up in patients with partial meniscal injuries. However, the existing literature lacks sufficient direct comparative studies between these two types of meniscal implants. Additionally, variations in study quality, follow-up duration, and patient characteristics further hinder a definitive assessment of their relative effectiveness [50]. Despite these limitations, CMI has demonstrated a favorable safety scaffold and long-term clinical efficacy, particularly in improving functional outcomes and reducing pain scores [7]. Additionally, clinical trial have demonstrated that PU-based scaffolds may offer superior results in patients with chronic meniscal injuries, often resulting from torsional injuries, when compared with those with acute injuries, which are more commonly observed in older individuals [43].

This study systematically evaluated the clinical outcomes associated with artificial meniscal implants, specifically CMI and PU-based scaffolds, in the treatment of partial meniscus injuries. The analysis compared patient-reported outcomes measures, including the VAS, IKDC score, Lysholm score, KOOS, and Tegner activity scores between the two implant types. These scores are crucial for assessing knee function and evaluating the clinical effectiveness of meniscal repair strategies. To ensure consistency and accuracy in evaluating clinical outcomes, follow-up periods were classified into short-term, mid-term, and long-term, in accordance with previous literature and established reporting standards [44, 51].

The analysis showed a decrease in VAS scores following treatment with both CMI and PU-based meniscal scaffolds during short-term and long-term follow-up. Importantly, the results did not show a significant difference in short-term follow-up VAS scores between CMI and PU-based scaffolds, suggesting comparable short-term clinical performance. However, a divergence was observed in the mid-term follow-up, where CMI demonstrated a greater average reduction in VAS scores (−5.63) compared with PU-based scaffolds (−1.97). This suggests that while both meniscal implant types are effective initially, CMI may offer superior pain relief in the longer term. The wide CI for CMI in the mid-term (−13.47 to 2.20), however, indicates variability in the data and warrants cautious interpretation. This analysis supports the use of both CMI and PU-based scaffolds for partial meniscus injuries, with evidence pointing to a potential advantage of CMI in mid-term pain reduction.

Using the IKDC score, the findings demonstrated a statistically significant improvement in knee function following treatment with both types of meniscal implants, as assessed across multiple follow-up periods. Notably, patients treated with PU-based scaffolds exhibited greater improvements in IKDC scores at both short-term and mid-term follow-up. This superior performance may be attributed to the favorable mechanical and biological properties of PU-based scaffolds. The consistent trend observed enhances the reliability of these findings and supports the hypothesis that scaffold composition, integration, and biomechanical support play critical roles in clinical outcomes. Moreover, these results are in agreement with previous literature [35].

The analysis showed significant improvements in Lysholm scores during short-term, mid-term, and long-term follow-up periods for both CMI and PU-based scaffolds. During the short-term and mid-term follow-ups, CMI demonstrated superior outcomes compared with PU-based scaffolds, which may reflect differences in tissue regeneration capacity or degradation rate of scaffold during the intermediate healing phase. However, at the long-term follow-up, PU-based scaffolds exhibited a markedly higher WMDs of 50.70 compared with 33.67 for CMI, suggesting that PU-based implants may provide more durable functional benefits over period. This sustained advantage could be attributed to the enhanced biomechanical resilience and prolonged structural support provided by PU materials during extended periods of joint loading.

The results indicated that both types of meniscal implants led to improvements in physical activity levels, as measured by Tegner scores for follow-up periods. However, CMI consistently outperformed PU-based scaffolds at each follow-up period. During the short-term follow-up, CMI was associated with a statistically significant increase in Tegner scores, with a mean difference of 2.02, reflecting early restoration of physical activity. In contrast, PU-based scaffolds demonstrated negligible improvement, suggesting limited short-term effectiveness in restoring pre-injury activity levels. At mid-term follow-up, both scaffold types were associated with increased activity levels, however, CMI showed a more pronounced effect compared with PU-based scaffolds, although the latter did achieve statistical significance. Long-term follow-up data further supported the superior functional recovery associated with CMI, with a mean improvement of 2.98 points, compared with 1.50 points for PU-based scaffolds. The sustained advantage of CMI may be attributed to its favorable biological properties, such as enhanced tissue compatibility and integration, which contribute to improved long-term mechanical performance and tolerance of physical activity. In contrast, the relatively modest long-term improvement observed with PU-based scaffolds, despite reaching statistical significance, suggests that their biomechanical or regenerative properties may be less effective in supporting higher levels of physical function over follow-up period.

This comprehensive meta-analysis of KOOS subscale scores provides valuable insights into patient-reported outcomes associated with both meniscal implants. CMI and PU-based scaffolds demonstrated clinically meaningful improvements across the KOOS domains including ADL, symptoms, sports, pain, and QOL. However, the patterns of recovery varied across subscales and follow-up periods. The analysis confirmed that KOOS score improved following treatment with both CMI and PU-based meniscal scaffolds. Notably, no significant difference was observed in pain score improvements between the two scaffold types across follow-up periods. For the Sports and QOL subscales, CMI showed greater improvements at mid-term follow-up, while PU-based scaffolds exhibited superior outcomes in short-term. A reversed trend was observed for the symptoms subscale, whereby PU-based scaffolds demonstrated greater short-term effectiveness, while CMI implants resulted in more substantial improvements at mid-term follow-up. CMI demonstrated significant improvement in symptoms scores at mid-term follow-up, while PU-based meniscal scaffolds were more effective for symptoms scores in the short-term. Overall, CMI were associated with greater improvements in ADL and sports function, whereas PU-based scaffolds demonstrated more consistent benefits in symptoms, pain, and QOL. The clinical efficacy of both implants is clear, but scaffold choice should be personalized based on period of effects and specific patient needs.

Although most outcome scores favored PU-based scaffolds over CMI, the differences in clinical scores for PU-based scaffolds were as follows: VAS (short-term) showed a mean difference of −1.98 (95% CI −2.60 to −1.37); Lysholm (long-term) improved by 50.70 points (95% CI 47.71–53.69); IKDC scores indicated a short-term difference of 31.25 points (95% CI −40.27 to −22.24) and a long-term difference of 33.00 points (95% CI 26.20–39.81); Symptoms (short-term) improved by 19.10 points (95% CI 12.54–25.65); Quality of Life (QOL) scores increased by 25.92 points (95% CI 20.50–31.34) in the short term and 31.38 points (95% CI 17.89–44.87) in the long term. This discrepancy may be attributed to the limited number of studies involving collagen implants included in the analysis. A recent systematic review by Han et al. highlighted that differences in study methodologies, patient populations, and defect details lead to inconsistent results about meniscal implants. Additionally, the lack of well-designed randomized trials and standardized data on key factors like defect size, location, and patient age restricts thorough subgroup analysis. As a result, there is no clear evidence favoring either collagen- or polyurethane-based scaffolds, which aligns with our findings. [52]. This current systematic review and meta-analysis has some limitations owing to the weaknesses of the included studies, such as heterogeneous follow-up periods for assessing efficacy or incomplete data and geographical and methodological disparities which may impact the overall conclusions. Despite the greater number of studies available on PU-based scaffolds compared with CMI, we minimized potential bias in pooled outcome estimates by applying the SMD and WMD approach. SMD allows comparison across studies with varying scales and sample sizes by standardizing the effect size, thereby reducing the influence of study number imbalance. Furthermore, the use of a random-effects model and sensitivity analyses helped account for heterogeneity and ensured the robustness of our findings. It is important to acknowledge that conducting subgroup meta-analyses on the basis of critical clinical variables, such as patient age, defect size, and defect location, would have provided valuable insights to guide clinicians in selecting the most appropriate scaffold for meniscal repair tailored to individual patient characteristics. Accordingly, one of the primary objectives of this study was to perform subgroup analyses stratified by defect location and defect size to explore potential sources of heterogeneity. However, this objective could not be realized owing to insufficient reporting of clinical outcomes stratified by these variables across the included studies, as presented in Table 4 (see Supplementary File 1). Specifically, only a single study reported outcomes stratified by tear resolution for the PU-based scaffold, while no comparable stratified data were available for the CMI. For subgroup meta-analyses to yield reliable and clinically meaningful conclusions, it is essential to have at least two, and preferably three or more, independent studies presenting consistent stratification and outcome reporting for each scaffold type. The absence of such data precluded the possibility of performing methodologically robust subgroup analyses. Consequently, any attempt to conduct these analyses under the current data limitations would risk generating misleading or inconclusive results, thereby failing to provide practical guidance for scaffold selection in partial meniscus injuries. This limitation highlights a critical gap in the current literature and underscores the need for future clinical studies to adopt standardized reporting protocols that include detailed stratification by relevant patient and defect characteristics. Such efforts would enhance the granularity of available evidence, enabling more personalized and evidence-based decision-making in meniscal scaffold selection. Additionally, the limited availability of artificial meniscal implants varies across different countries, which may impact the results. However, it is important to emphasize that this systematic review and meta-analysis was prepared on the basis of the available data to assist physicians in the treatment of partial meniscus injuries. In following, the review conducted a thorough search strategy across multiple databases and included a larger number of studies compared with previous systematic reviews. Unlike prior reviews that examined studies with diverse designs, this analysis specifically evaluated the use of CMI and PU-based meniscal implants, assessing a comprehensive set of clinical and functional outcomes. Good clinical outcomes, few complications, and low failure rates have been observed for two types of meniscal implants.

Overall, both CMI and PU-based scaffolds demonstrated improvement in clinical and functional outcomes across the included studies. While some studies reported more favorable outcome scores with PU-based scaffolds compared with CMI, these findings should be interpreted with caution due to the lack of direct comparative trials. PU-based scaffolds are designed to provide mechanical support to the knee joint while slowly degrading and being replaced by regenerated tissue within 5 years [35]. Their synthetic composition may facilitate faster integration, potentially contributing to short-term clinical effectiveness. PU-based scaffolds enhance clinical outcomes for patients with partial meniscal defects by supporting cellular ingrowth and tissue regeneration. Additionally, PU-based implant have shown promising results in supporting survival rates and tissue regeneration in patient with partial meniscus injuries. Some studies have also reported survival rates comparable to meniscal allograft transplantation following total meniscectomy, with positive outcomes maintained up to 5 years post-implantation. These characteristics suggest that PU-based scaffolds may be beneficial in cases where rapid recovery and short-term symptom relief, such as in patients with chronic meniscal injuries [53]. In contrast, CMI, which is a porous matrix composed of type I collagen and GAG, promotes gradual tissue regeneration, and offers long-term stability while improving functionality. Additionally, CMI emphasizes the regeneration of lost meniscal tissue, supports sustained tissue regeneration, and has demonstrated promising results in enhancing functional outcomes and pain scores over an extended follow-up period. CMI is more suitable for cases where long-term functional restoration is prioritized, such as in younger patients or those requiring gradual tissue regeneration. However, the lack of direct comparative studies between CMI and PU-based scaffolds makes it challenging to definitively assess their relative effectiveness. Therefore, further research is needed to address this gap.

## Conclusions

This systematic review and meta-analysis aimed to evaluate the clinical effectiveness of CMI and PU-based scaffolds for the treatment of partial meniscal injuries. The key findings indicate that both scaffold types significantly improve pain and functional outcomes across short-, mid-, and long-term follow-up periods. PU-based scaffolds offer faster integration and short-term effectiveness, while CMI promotes gradual tissue regeneration and long-term stability. While no statistically significant differences in overall clinical outcomes were observed between the two scaffold types, each demonstrated distinct functional advantages, such as faster symptomatic relief with PU-based scaffolds and more gradual regeneration with long-term stability using CMI that may be clinically relevant depending on patient-specific factors such as age, defect location, and treatment goals. The lack of direct comparative studies and variability in study quality preclude definitive conclusions regarding their relative superiority. Clinically, both implants appear to be safe and effective options for meniscal repair, but further high-quality, comparative research is needed to guide optimal scaffold selection and long-term management strategies.

## Supplementary Information

Supplementary material 1

## Data Availability

All data generated or analyzed during this study are included in this published article.

## Abbreviations

CMI: Collagen meniscus implant
PU-based: Polyurethane-based
VAS: Visual analog scale
IKDC: International knee documentation committee
KOOS: Knee injury and osteoarthritis outcome
ADL: Activities of daily living
QOL: Quality of life
GAG: Glycosaminoglycan
PCL: Polycaprolactone
PCU: Polycarbonate urethane
UHMWPE: Ultrahigh molecular weight polyethylene
SMD: Standardized mean differences
WMD: Weighted mean difference
CI: Confidence interval
NIH: National institutes of health
