# Supplementary material for: Clinical outcomes of artificial meniscus scaffolds for partial meniscus injury: a systematic review and meta-analysis
Source: Knee Surg Relat Res. 2025 Sep 30;37:41. doi: 10.1186/s43019-025-00293-2 (PMC12486669; doi:10.1186/s43019-025-00293-2)
Supplement: Supplementary file 1 — Supplementary material 1 [file 43019_2025_293_MOESM1_ESM.docx]

**Supplementary File 1**

**Clinical Outcomes of Artificial Meniscus Scaffolds for Partial Meniscus Injury: A Systematic Review and Meta-Analysis**

**Short title**: Clinical Outcomes of Artificial Meniscus Scaffolds.

**Table 1.** The main characteristics of the included studies.

| **Author** | **Country** | **Study Design** | **Sex (M/F)** | **Age**  (Mean ± SD) | **BMI**  (Mean ± SD) | **Sample Size** | **Defect Size** (Mean ± SD) (mm) | **Scaffold Size** (Mean ± SD) (mm) | **Number of Defect Site** (Lateral/Medial/ Patella/Trochlea) | **Type of Scaffolds** | **Follow-up**  Range: (Min-Max) |
| --- | --- | --- | --- | --- | --- | --- | --- | --- | --- | --- | --- |
| Akkaya, M. [23] | Turkey | Pre-post | 20/0 | 32.2 ± 8.8 | 26.2 ± 4.2 | 20 | - | - | - | PU | 8 Y |
| Bulgheroni, E. [24] | Italy | NRIS | 13/4 | 32.9 ± 10.3 | NA | 17 | - | - | 0/17/0/0 | CMI | 10Y |
| Bulgheroni, E. [25] | Belgium | NRIS | 19/9 | 38.7 ± 9.7 | NA | 28 | - | PU: 45 ± 9.7,  CMI: 43 ± 1.17 | 0/28/0/0 | CMI, PU | 1-2Y |
| Condello, Vin. [26] | Italy | Pre-post | 48/19 | 40.8 ± 10.6 | 25.4 ± 4.3 | 67 | - | - | 13/54/0/0 | PU | 3Y |
| De Coninck, T. [27] | Belgium | Pre-post | 12/12 | 35 | NA | 24 | - | 43.2 ± 12 | 8/18/0/0 | Actifit (PU) | 2Y |
| Dhollander, Aad. [28] | Belgium | NRIS | 24/20 | 32.13 (17-50) | 35 | 44 | 45.51 ± 10.25 | - | 15/29/0/0 | PU | 2-5Y |
| Efe, T. [29] | Germany | Pre-post | 8/2 | 29 (18-45) | 26 (23-30) | 10 | - | 39.2 ± 3.6 | - | Actifit (PU) | 6M-1Y |
| Filardo, Giu. [30] | Italy | Case series | 9/7 | 45 ± 13 | 25 ± 3 | 16 | - | - | 4/12/0/0 | PU | 2-6Y |
| Gelber, P. E. [31] | Spain | NRIS | 19/11 | 45.1 ± 8.3 | 26.2 ± 2.7 | 30 | - | 40.3 ± 6 | 0/30/0/0 | Actifit (PU) | 2.5Y |
| Gelber, P. E. [31] | Spain | Pre-post | 12/42 | 40.2 | 25.7 ± 4.4 | 64 | - | 42.7 ± 7.5 | 14/40/0/0 | Actifit (PU) | 5Y |
| Gelber, P. E. [32] | Spain | Pre-post | 46/16 | 41/3 | NA | 62 | - | - | - | PU | 2Y |
| Hirschmann, M. T. [33] | Switzerland | Pre-post | 47/20 | 36 ± 10 | NA | 60 | - | - | 12/55/0/0 | CMI | 6M-2Y |
| Kon, E. [34] | Italy | NRIS | 9/21 | 29/3 | NA | 27 | - | - | 5/13/0/0 | CMI | 6M-2Y |
| Leroy, A.[35] | France | Pre-post | 8/7 | 30 (19-47) | 25/5 | 15 | - | 37 ± 3.25 | 9/6/0/0 | Actifit (PU) | 1-5Y |
| Martín-Hernández, C. [36] | Spain | Pre-post | 6/4 | 30.6 (16-50) | 24/5 | 10 | - | - | 1/9/0/0 | PU | 6M-3Y |
| Miroslav Haspl [37] | Croatia | Pre-post | 5/4 | 36 ± 7.7 | 26.7 ± 4.67 | 9 | - | 46.1 ± 6.25 | 4/5/0/0 | Actifit (PU) | 32.5 (0–68)M |
| Monllau, J. C. [38] | Spain | Pre-post | 20/5 | 29.2 (18.3-48.2) | 25.1 (18 - 44.1) | 25 | - | 48.2 ± 8.754 | 0/5/0/0 | CMI | 1Y |
| Monllau, J. C. [39] | Spain | Pre-post | 25/7 | 41.3 ± 11.1 | NA | 32 | - | 45 ± 7.6 | 11/21/0/0 | PU | 5Y |
| Schenk, L. [40] | Switzerland | Pre-post | 30/9 | 34 ± 10 | NA | 39 | - | 48 ± 11 | 7/32/0/0 | CMI | 1-7Y |
| Schüttler, K. F. [41] | Germany | Pre-post | NA | 32.5 ± 8 | NA | 18 | - | 45 ± 6.5 | - | Actifit (PU) | 6M-2Y |
| Schüttler, K. F. [42] | Germany | Pre-post | 13/6 | 35.6 ± 8.3 | 27.8 ± 3.5 | 23 | - | - | 3/13/5/2 | CMI | 6M-5Y |
| Toanen, C. [13] | France | Pre-post | 109/46 | 33.7 ± 10.4 | NA | 114 | 3.94 ± 10.5 | - | 54/101/0/0 | PU | 2-5Y |
| Verdonk, P. [43] | Belgium | Pre-post | 39/13 | 30.8 ± 9.4 | NA | 52 | - | - | 18/34/0/0 | PU | 6M-2Y |
| Zaffagnini, S. [44] | Italy | NRIS | 37/0 | 38 ± 14 | 25.2 ± 1.6 | 17 | 36 ± 9 | - | 0/17/0/0 | CMI | 5-10Y |
| Zaffagnini, S. [45] | Italy | Pre-post | 20/4 | 36.3 ± 11.5 | 23.8 ± 2.6 | 24 | 4.52 ± 0.81 | 45.8 ± 7.4 | 17/2/0/0 | CMI | 6M-2Y |
| Zaffagnini, S. [46] | Spain | Pre-post | 30/13 | 30.1 ± 12.0 | 24.3 ± 3.4 | 43 | 62 ± 13 | 46 ± 11 | 24/0/0/0 | CMI | 6M-2Y |

NRIS: non-randomized interventional study; NA: not applicable; CMI: collagen meniscus implant; PU: polyurethane; Y: year; M: month.

**Table 2.** The clinical outcomes evaluated in the included studies

| **Author** | **Type of Scaffolds** | **Pre-clinical outcomes** | **Post-clinical outcomes**  **(Short term)** | **Post-clinical outcomes**  **(Mid-term)** | **Post-clinical outcomes**  **(Long term)** |
| --- | --- | --- | --- | --- | --- |
| Akkaya, M. [23] | PU | Lysholm: 30.8 ± 4.3 | - | - | Lysholm: 81.5 ± 5.3 |
| Bulgheroni, E. [24] | CMI | Lysholm: 57.3 ± 16.9  Tegner: 3 ± 0.7 | - | - | Lysholm: 94.1 ± 8.2  Tegner: 6 ± 0.2 |
| Bulgheroni, E. [25] | CMI | Lysholm: 58.4 ± 17.3 | Lysholm: 94.5 ± 6 | - | - |
| Bulgheroni, E. [25] | PU | Lysholm: 67 ± 15.7 | Lysholm: 90.3 ± 13.1 | - | - |
| Condello, Vin. [26] | PU | IKDC: 72.5 ± 20.8  Lysholm: 53 ± 29  Tegner: 3 ± 1.7  Symptoms: 60.7 ± 31.2  ADL: 69.1 ± 31.5  Sports: 25 ± 25  Pain: 55.6 ± 31.7  QOL: 25 ± 21.7 | - | IKDC: 36.8 ± 25  Lysholm: 85 ± 30.2  Tegner: 5 ± 2.5  Symptoms: 85.7 ± 36.2  ADL: 94.1 ± 33.7  Sports: 75 ± 25  Pain: 91.7 ± 32  QOL: 75 ± 25 | - |
| De Coninck, T. [27] | Actifit (PU) | VAS: 5.4 ± 2.2  IKDC: 39.1 ± 18.2  Lysholm: 52.1 ± 17.4  Symptoms: 53.5 ± 19.6  ADL: 39.1 ± 18.2  Sports: 20.1 ± 22.1  Pain: 53.2 ± 21.02  QOL: 30 ± 18 | VAS: 2.1 ± 2.2  IKDC: 64.1 ± 24.6  Lysholm: 76.2 ± 20.1  ADL: 64.1 ± 24.6  Sports: 52.1 ± 28.4  Pain: 74.56 ± 23.89  QOL: 51.9 ± 28.8 | Symptoms: 77.2 ± 20.1 | - |
| Dhollander, Aad. [28] | PU | VAS: 56.2 ± 21.6  IKDC: 38.7 ± 14.8  Symptoms: 52.4 ± 19.7  ADL: 54.4 ± 21.5  Pain: 48.3 ± 20.3  Sport: 19.1 ± 20  QOL: 32.2 ± 14.2 | IKDC: 63.4 ± 24.3  ADL: 63.4 ± 24.3  Symptoms: 73.3 ± 18.4  Sports: 57 ± 35.6  Pain: 72.9 ± 23.6  QOL: 49.6 ± 14.2 | VAS: 19.3 ± 26.9  IKDC: 66.9 ± 23.1  Symptoms: 69.4 ± 20.9  ADL: 80.2 ± 26.1  Pain: 77.2 ± 24.5  Sports: 49.7 ± 34.8  QOL: 59.9 ± 24 | - |
| Efe, T. [29] | Actifit (PU) | VAS: 4.2 ± 1.6  Symptoms: 60.8 ± 18.9  ADL: 53.7 ± 6.3  Sports: 29.5 ± 12.8  Pain: 45.7 ± 22.7  QOL: 27.6 ± 19.1 | VAS: 2 ± 1.2  Symptoms: 85.9 ± 25.1  ADL: 90 ± 26.5  Sports: 79 ± 23.6  Pain: 82.5 ± 41.2  QOL: 70.8 ± 22.8 | - | - |
| Filardo, Giu. [30] | PU | Tegner: 2 ± 1.2  ADL: 45.6 ± 17.5 | Tegner: 3 ± 0.5  ADL: 75.3 ± 14.8 | IKDC:75 ± 16.8 | Tegner: 3.5 ± 0.7 |
| Gelber, P. E. [31] | Actifit (PU) | VAS: 7.9 ± 1  IKDC: 19.1 ± 5.9  ADL: 19.1 ± 5.9 | VAS: 2.1 ± 1.9  IKDC: 69.4 ± 25  ADL:69.4 ± 25 | - | - |
| Gelber, P. E., [31] | PU | VAS: 7.2 ± 1.1  IKDC: 20.1 ± 4.4  ADL: 20.1 ± 4.4 | VAS: 2.5 ± 2.1  IKDC: 76.8 ± 15  ADL: 76.8 ± 15.3 | - | - |
| Gelber, P. E. [31] | Actifit (PU) | VAS: 7.5 ± 1.2  IKDC: 32.5 ± 11.1 | IKDC: 78.2 ± 14.8 | VAS: 2.3 ± 1.5  IKDC: 78.2 ± 14.8 | - |
| Gelber, P. E. [32] | PU | VAS: 7.22 ± 1.22  Tegner: 5.1 ± 1.8 | VAS: 2.67 ± 2.1  Tegner: 4 ± 1.6 | - | - |
| Hirschmann, M. T. [33] | CMI | VAS: 4.4 ± 2.2  Lysholm: 68 ± 20 | VAS: 2.2 ± 2.1  Lysholm: 93 ± 9 | - | - |
| Kon, E. [34] | CMI | IKDC: 47.3 ± 17.5  ADL: 47.3 ± 17.5 | IKDC: 74.6 ± 15.3  ADL: 74.6 ± 15.3 | - | - |
| Leroy, A.[35] | Actifit (PU) | VAS: 5.5 ± 2  IKDC: 51.2 ± 20  Symptoms: 69.4 ± 13  ADL: 51.2 ± 20  Sports: 51.2 ± 14  Pain: 62.9 ± 15  QOL: 40.9 ± 18 | VAS: 2.9 ± 2.1  IKDC: 65.1 ± 22  Symptoms: 76.5 ± 15  ADL: 65.1 ± 22  Sports: 61.2 ± 17  Pain: 77.8 ± 18  QOL: 64.3 ± 25 | VAS: 2.9 ± 2.6  IKDC: 66.1 ± 23  Symptoms: 68.3 ± 23  ADL: 81.7 ± 23  Sports: 53.5 ± 33  Pain: 76.1 ± 25  QOL: 59.9 ± 31 | - |
| Martín-Hernández, C. [36] | PU | VAS: 5.7 ± 2.4  Lysholm: 63.5 ± 22.1 | VAS: 1.9 ± 1.6  Lysholm: 83.3 ± 24.5 | VAS: 1.9 ± 1.6  Lysholm: 84.4 ± 25.8 | - |
| Miroslav Haspl [37] | Actifit (PU) | VAS: 7.7 ± 1  Lysholm: 61.7 ± 9 | VAS: 3.1 ± 0.5  Lysholm: 86.4 ± 8.25 | - | - |
| Monllau, J. C. [38] | CMI | VAS: 5.5 ± 1.5  Lysholm: 59.9 ± 15 | VAS: 1.5 ± 1.2  Lysholm: 89.6 ± 5.5 | - | Lysholm: 87.56 ± 10.25 |
| Monllau, J. C. [39] | PU | IKDC: 41.7 ± 20.8  Lysholm:40.7 ± 20.3  Tegner: 5.1 ± 2.5 | - | IKDC: 79.4 ± 10.6  Lysholm: 78.1 ± 39  Tegner: 5.7 ± 2.8 | - |
| Schenk, L. [40] | CMI | VAS: 4.3 ± 3.2  Lysholm: 66 ± 20  Tegner: 3.5 ± 2.2 | VAS: 0.8 ± 1.4  Lysholm: 95 ± 6.5  Tegner: 6 ± 3.2 | - | Lysholm: 91 ± 8  Tegner: 6 ± 3.2 |
| Schüttler, K. F. [41] | Actifit (PU) | VAS: 5.1 ± 2  Symptoms: 60 ± 16.2  ADL: 53 ± 16  Sports: 26 ± 20.5  Pain: 47 ± 14.5  QOL: 33.9 ± 19.3 | VAS: 5.1 ± 2.1  Symptoms: 81 ± 13.4  ADL: 91 ± 14.7  Sports: 66 ± 28.5  Pain: 83 ± 18.6  QOL: 63 ± 18.9 | - | - |
| Schüttler, K. F. [42] | CMI | VAS: 5.88 ± 2.15  IKDC: 56.5 ± 8.4  Symptoms: 48.3 ± 10.7  ADL: 56.5 ± 8.4  Sports: 31.3 ± 19.2  Pain: 49.79 ± 16.33  QOL: 32.4 ± 11.8 | VAS: 2.4 ± 2.1  IKDC: 68.1 ± 12.4  Symptoms: 51.7 ± 15.4  ADL: 68.1 ± 12.4  Sports: 63.8 ± 29.1  Pain: 75.11 ± 16.79  QOL: 53.5 ± 24.7 | VAS: 2.14 ± 2.2  IKDC: 70.8 ± 22.3  Symptom: 72.9 ± 25  Sport: 68.2 ± 26.5  Pain: 76.59 ± 25.28  QOL: 59.3 ± 30 | - |
| Toanen, C. [13] | PU | VAS: 54 ± 20.7  IKDC: 41.8 ± 16.3  Lysholm: 60.5 ± 19.6  Symptoms: 56 ± 19.7  ADL: 41.8 ± 16.3  Sports: 28.5 ± 24  Pain: 54.2 ± 22  QOL: 30.7 ± 16.7 | VAS: 23.7 ± 17.2  IKDC: 71.5 ± 19  Lysholm: 88.1 ± 14.3  Symptoms: 83 ± 15.6  ADL: 71.5 ± 19  Sports: 61.3 ± 26.6  Pain: 78.8 ± 17.4 | VAS: 15.2 ± 19.2  IKDC: 72.3 ± 21.4  Lysholm: 84.5 ± 20.1  Symptoms: 78.2 ± 9.5  ADL: 82.1 ± 21  Sports: 53.9 ± 31.4  Pain: 78.4 ± 21.3  QOL: 56.2 ± 26.4 | - |
| Verdonk, P. [43] | PU | VAS: 45.7 ± 26.2  IKDC: 45.4 ± 17.8  Lysholm: 60.1 ± 19.2  Symptoms: 64.6 ± 22.3  ADL: 45.4 ± 17.8  Sports: 30.5 ± 28.7  Pain: 57.5 ± 22.2  QOL: 33.9 ± 19.3 | VAS: 20.3 ± 23.5  IKDC: 70.1 ± 23  Lysholm: 80.7 ± 19.5  Symptoms: 78.3 ± 18.5  ADL: 70.1 ± 23  Sports: 59 ± 33.4  Pain: 78.6 ± 22.5  QOL: 56.6 ± 24.2 | - | - |
| Zaffagnini, S. [44] | CMI | VAS: 6 ± 0.5  Lysholm: 47.5 ± 4.4  Tegner: 1 ± 0.6 | Tegner: 4 ± 0.2 | VAS: 1.6 ± 0.4  Lysholm: 93.6 ± 5 | Lysholm: 92 ± 5.7  Tegner: 4 ± 0.7 |
| Zaffagnini, S. [45] | CMI | VAS: 55.2 ± 29.4  Lysholm: 64 ± 16.2  Tegner: 3 ± 1.2 | VAS: 1.9 ± 2.5  Lysholm: 92.7 ± 13.8  Tegner: 5 ± 0.7 | - | - |
| Zaffagnini, Ste. [46] | CMI | Lysholm: 64.3 ± 18.4  Tegner: 3 ± 0.5 | Lysholm: 93.2 ± 7.2  Tegner: 5 ± 0.7 | - | - |

CMI: collagen meniscus implant; PU: polyurethane; VAS: visual analog scale; IKDC: international knee documentation committee; ADL: knee injury and osteoarthritis outcome; QOL: Quality of life; short-term: 2.5 – 5 years; mid-term: 6 months to 2.5 years; long-term: 10 years.

**Table 3.** The quality assessment finding of included studies

| **No.** | **Study** | **Q1** | **Q2** | **Q3** | **Q4** | **Q5** | **Q6** | **Q7** | **Q8** | **Q9** | **Q10** | **Q11** | **Q12** | **Quality rating** |
| --- | --- | --- | --- | --- | --- | --- | --- | --- | --- | --- | --- | --- | --- | --- |
| 1 | Akkaya, M. [23] | Yes | Yes | Yes | NR | Yes | Yes | Yes | No | NR | Yes | Yes | NA | Good |
| 2 | Bulgheroni, E. [24] | Yes | Yes | Yes | NR | Yes | Yes | Yes | No | Yes | Yes | Yes | NA | Good |
| 3 | Bulgheroni, E. [25] | Yes | Yes | Yes | Yes | Yes | Yes | No | Yes | Yes | Yes | Yes | NA | Good |
| 4 | Condello, Vin. [26] | Yes | Yes | Yes | Yes | Yes | Yes | Yes | No | No | Yes | Yes | NA | Good |
| 5 | De Coninck, T. [27] | Yes | Yes | No | Yes | No | Yes | Yes | No | Yes | Yes | Yes | NA | Good |
| 6 | Dhollander, Aad. [28] | Yes | Yes | Yes | Yes | Yes | Yes | No | Yes | No | Yes | Yes | NA | Good |
| 7 | Efe, T. [29] | Yes | Yes | No | No | No | Yes | Yes | No | NR | Yes | Yes | NA | Fair |
| 8 | Filardo, Giu. [30] | Yes | Yes | No | Yes | No | Yes | Yes | No | NR | Yes | Yes | NA | Good |
| 9 | Gelber, P. E. [31] | Yes | No | Yes | Yes | Yes | Yes | Yes | No | Yes | Yes | Yes | NA | Good |
| 10 | Gelber, P. E. [31] | Yes | Yes | Yes | Yes | Yes | Yes | Yes | No | No | No | No | NA | Fair |
| 11 | Gelber, P. E. [32] | Yes | No | Yes | Yes | Yes | No | No | Yes | NR | Yes | No | NA | Poor |
| 12 | Hirschmann, M. T. [33] | Yes | No | No | Yes | Yes | Yes | No | No | NR | Yes | Yes | NA | Fair |
| 13 | Kon, E. [34] | Yes | Yes | No | Yes | No | Yes | Yes | No | NR | Yes | Yes | NA | Good |
| 14 | Leroy, A.[35] | Yes | Yes | Yes | No | No | Yes | Yes | No | NR | Yes | Yes | NA | Good |
| 15 | Martín-Hernández, C. [36] | Yes | Yes | No | No | No | Yes | Yes | No | No | No | Yes | NA | Fair |
| 16 | Miroslav Haspl [37] | Yes | No | No | Yes | No | No | No | No | Yes | Yes | Yes | NA | Poor |
| 17 | Monllau, J. C. [38] | Yes | Yes | Yes | NR | No | Yes | Yes | No | NR | Yes | Yes | NA | Fair |
| 18 | Monllau, J. C. [39] | Yes | Yes | Yes | Yes | Yes | Yes | Yes | No | Yes | Yes | Yes | NA | Good |
| 19 | Schenk, L. [40] | Yes | No | No | Yes | Yes | No | Yes | Yes | NR | Yes | Yes | NA | Good |
| 20 | Schüttler, K. F. [41] | Yes | Yes | Yes | Yes | Yes | No | Yes | No | NR | Yes | Yes | NA | Good |
| 21 | Schüttler, K. F. [42] | Yes | No | Yes | Yes | Yes | Yes | Yes | Yes | No | Yes | Yes | NA | Good |
| 22 | Toanen, C. [13] | Yes | Yes | Yes | Yes | Yes | Yes | Yes | No | No | Yes | Yes | NA | Good |
| 23 | Verdonk, P. [43] | Yes | Yes | No | Yes | Yes | No | Yes | Yes | No | Yes | Yes | NA | Good |
| 24 | Zaffagnini, S. [44] | Yes | Yes | Yes | Yes | No | Yes | Yes | Yes | Yes | Yes | Yes | NA | Good |
| 25 | Zaffagnini, S. [45] | Yes | Yes | No | Yes | No | Yes | Yes | Yes | NR | Yes | Yes | NA | Good |
| 26 | Zaffagnini, S [46] | Yes | Yes | Yes | Yes | Yes | Yes | Yes | No | Yes | Yes | Yes | NA | Good |

NA: not applicable, NR: not reported

**Table 4.** The defect’s data.

| Author | Sample Size | Right/Left | PU-Based Scaffolds | | | | | CMI | | | | | | | | |
| --- | --- | --- | --- | --- | --- | --- | --- | --- | --- | --- | --- | --- | --- | --- | --- | --- |
|  |  |  | Defect Size (mm) | Scaffold Size (mm) | Defect Location | | | Defect Size (mm) | Scaffold Size  (mm) | | | Defect Location | | | | |
|  |  |  |  |  | Lateral | Medial | Other^#^ |  |  |  |  | Lateral | | Medial | | Other^#^ |
| Akkaya, M. [23] | 20 | 16/4 | – | – | – | – | – | – | | – | | – | | – | | – |
| Bulgheroni, E. [24] | 17 | – | – | – | – | – | – | – | | – | | – | | 17 | | – |
| Bulgheroni, E. [25] | 28 | 20/8 | – | 45 ± 9.7 | – | 28* | – |  | | 43 ± 1.17 | | 28* | | – | | – |
| Condello, Vin. [26] | 67 | – | – | – | 13 | 54 | – | – | | | – | – | | – | | – |
| De Coninck, T. [27] | 24 | – | – | 43.2 ± 12 | 8 | 18 | – | – | | | – | – | | – | | – |
| Dhollander, Aad. [28] | 44 | – | 45.5 ± 10.25 | – | 15 | 29 | – | – | | | – | – | | – | | – |
| Efe, T. [29] | 10 | – | – | 39.2 ± 3.6 | 0 | 10 | – | – | | | – | – | | – | | – |
| Filardo, Giu. [30] | 16 | – | – | - | 4 | 12 | – | – | | | – | – | | – | | – |
| Gelber, P. E. [31] | 30 | 30/30 | – | 40.3 ± 6 | - | 30 | – | – | | | – | – | | – | | – |
| Gelber, P. E. [31] | 64 | 23/31 | – | 42.7 ± 7.5 | 14 | 40 | – | – | | | – | – | | – | | – |
| Gelber, P. E. [32] | 62 | – | – | – | – | – | – | – | | | – | – | | – | | – |
| Hirschmann, M. T. [33] | 60 | – | – | – | – | – | – | – | | | Medial: 7.5-9, Lateral: 9.5** | 12 | | 55 | | – |
| Kon, E. [34] | 27 | – | – | - | 5 | 13 | – | – | | | – | – | | – | | – |
| Leroy, A. [35] | 15 | – | – | 37 ± 3.25 | 9 | 6 | – | – | | | – | – | | – | | – |
| Martín-Hernández, C. [36] | 10 | – | – | – | 1 | 9 | – | – | | | – | – | | – | | – |
| Miroslav Haspl [37] | 9 | – | – | 46.1 ± 6.25 | 4 | 5 | – | – | | | – | – | | – | | – |
| Monllau, J. C. [38] | 25 | – | – | – | – | – | – | 48.2 ± 8.75 | | | - | 5 | | – | | – |
| Monllau, J. C. [39] | 32 | 14/18 | – | – | 11 | 21 | – | – | | | – | – | | – | | – |
| Schenk, L. [40] | 39 | – | – | – | – | – | – | 48 ± 11 | | | 7 | 32 | | – | | – |
| Schüttler, K. F. [41] | 18 | – | 45 ± 6.5 | – | – | – | – | – | | | – | – | – | | | |
| Schüttler, K. F. [42] | 23 | – | – | – | – | – | – | 3.7 ± 1.9*** | | | – | 3 | 13 | | 7 | |
| Toanen, C. [13] | 114 | – | 39.4 ± 10.5 | – | 54 | 101 | – | – | | | – | – | – | | – | |
| Verdonk, P. [43] | 52 | 25/27 | 21-defects: (40-50),  12-defects: (50 -60),  10-defects: (> 60) | – | – | 34 | – | – | | | – | – | – | | – | |
| Zaffagnini, S. [44] | 17 | – | – | – | – | – | 36±9 | – | | | – | 17 | 36±9 | | – | |
| Zaffagnini, S. [45] | 24 | 15/9 | – | – | – | – | 45.2±8.1 | 45.8±7.4 | | | 2 | 17 | 45.2±8.1 | | – | |
| Zaffagnini, S. [46] | 43 | 24/19 | – | – | – | – | 62±13 | 46±11 | | | 24 | - | 62±13 | | – | |

CMI: collagen meniscus implant; PU: polyurethane

(#): Patella or Trochlea locations, (-): not applicable, (*) The site of the defect was not reported separately for PU-based or CMI defect, (**) The width of the implant was reported in millimeters (mm), (***) The scaffold area was reported in square centimeters (cm^2^).

Note: This table provides a detailed overview of the key variables-patient age, defect size, and defect location-reported in the included studies, categorized separately for PU-based and CMI. As highlighted in the Discussion section, it is important to note that clinical outcomes stratified by these variables (size, location, and patient age) were generally not reported separately across the studies. This lack of stratified outcome data limits the ability to perform subgroup analyses and hinders a more nuanced understanding of how these factors may influence scaffold performance and patient prognosis. Consequently, while the table summarizes the demographic and defect characteristics, it underscores the current gap in the literature regarding the impact of these variables on clinical efficacy and highlights the need for future research to incorporate standardized, detailed reporting to facilitate more personalized scaffold selection and treatment planning.
